# Development and prevalence of breastfeeding initiation in a tertiary obstetric center and its influencing factors

**DOI:** 10.1186/s13006-025-00717-5

**Published:** 2025-04-03

**Authors:** A. L. Biermann, L. Steinkasserer, L. Radomsky, C. von Kaisenberg, P. Hillemanns, Lars Brodowski

**Affiliations:** Department of Gynaecology and Obstetrics, Hannover Medical School, Carl-Neuberg-Straße 1, 30625 Hannover, Germany

**Keywords:** Tertiary obstetric center, Breastfeeding, Initiation

## Abstract

**Background:**

Tertiary obstetric centers are responsible for the care of all their newborns and for supporting mothers during breastfeeding. The aim of this work is to analyze the development and prevalence of breastfeeding initiation in a tertiary obstetric center. Subsequently, factors influencing the initiation of breastfeeding will be investigated.

**Methods:**

This is a retrospective study collecting data of all births of a non-selected cohort from 2017 to 2022 of singleton pregnancies at the Medical School of Hannover, Germany. Retrospective data of 16,092 women were used. We examined type of infant nutrition in our maternity unit in mothers by self-report, which was a routine survey conducted by a breastfeeding and lactation consultant within the framework of the perinatal quality assurance initiative. Secondly, factors impacting breastfeeding initiation were investigated (maternal BMI, gestational age, parity, special risk factors and birth mode) using a second cohort of 4,603 mother-child-pairs of live born, singleton full-term newborns.

**Results:**

Over the observed period, the rate of ever breastfeeding women was 93% in 2017 and 83% in 2022 indicating decreased adherence to breastfeeding. The rate of exclusively breastfeeding at the breast decreased by 21% over observed period (from 78 to 57%). While the group of feeding infant formula only and breastfeeding cessation before discharge remained stable, the rate of supplementary feeding, and breastfeeding and feeding infant formula, increased significantly. The rate of exclusive breastfeeding at the breast was lower in the groups of obese compared to normal-weight women (59.1% vs. 78.2%), women undergoing a cesarean section in comparison to vaginal birth (62.3% vs. 78.1%) and deliveries at 38 weeks of gestation compared to 40 weeks of gestation (62.7% vs. 77.3%). The infants of women with diabetes mellitus (74.2% vs. 62%) or gestational diabetes (74% vs. 65%) were significantly more likely to require infant formula than those without risk factors.

**Conclusions:**

Those women with the potentially highest benefit of breastfeeding to not exert their potential for risk reduction. Adequate awareness among healthcare professionals is imperative to capitalize on the brief but substantial opportunity to influence breastfeeding behavior in a tertiary obstetric center.

**Supplementary Information:**

The online version contains supplementary material available at 10.1186/s13006-025-00717-5.

## Background

The World Health Organization (WHO) recommends infants to be exclusively breastfed for the first six months of life. After these six months, breastfeeding should be continued in combination with appropriate complementary foods until the age of two years or beyond, if this is desirable for both mother and child [1].

At the European level, the European Society for Paediatric Gastroenterology, Hepatology, and Nutrition (ESPGHAN) has taken positions on infant nutrition recommendations [2, 3]. Whereas in Germany, there is a joint recommendation on infant nutrition from the German Society for Pediatric and Adolescent Medicine and the federally funded network “Healthy Start in Life” [4]. Here, almost 90% of mothers intend to breastfeed their child after birth. 97% of these mothers try to breastfeed after birth [5, 6].

Approximately 80% worldwide breastfeed their newborns in the first few weeks after birth. However, this rate decreases over time. After about six months, around 50% of mothers are still breastfeeding exclusively or partially [5].

The medical facilities where the delivery took place are the first point of contact for the mother, her newborn and their families and play a key role in supporting breastfeeding initiatives. Factors related to hospital care have a significant influence on breastfeeding [7–10]. There is also evidence that sociodemographic characteristics of mothers influence breastfeeding [11, 12].

The opportunity to influence breastfeeding behavior through a tertiary obstetric center is brief but significant. This applies to the period before birth, as part of preparatory discussions with the expectant mother, as well as the period immediately peri- and postpartum. When education during pregnancy is lacking, the importance of in-hospital information and support is even more crucial [13]. The postpartum period represents a pivotal opportunity to lay the foundations for breastfeeding. A systematic review showed that breastfeeding support from professionals before, during and after childbirth significantly increases the duration of exclusive breastfeeding [14]. The promotion of breastfeeding immediately after birth is of critical importance for the successful initiation and duration of breastfeeding [15, 16]. A further key role for the tertiary obstetric center is to enhance parents’ knowledge regarding significant aspects of their children’s nutrition [17]. In addition, the promotion of exclusive breastfeeding represents one of the most cost-effective public health interventions currently available [16]. The most important factors for exclusive breastfeeding are breastfeeding information, early breastfeeding initiation and alternative feeding methods. Individual support after the inpatient stay is of great importance for long-term breastfeeding success [18]. Tertiary obstetric centers are responsible for the care of all newborns and for supporting mothers during breastfeeding. This includes healthy, full-term neonates, but also premature babies and malformed or severely ill fetuses. In view of the special challenges that mothers often experience in these settings, it is of great importance to examine the practices and approaches to promoting breastfeeding in such facilities.

The aim of this work is to analyze the development and prevalence of breastfeeding initiation in a tertiary obstetric center. Subsequently, factors influencing the initiation of breastfeeding will be investigated. On this basis, the needs of mothers and infants are to be recorded and possible potential for improvement is to be identified. A systematic consideration of these aspects is intended to contribute to optimizing breastfeeding promotion in the early postnatal phase.

## Methods

### Study population

The study population consists of two cohorts. (I) First a retrospective data analysis of all births of a non-selected cohort from 2017 to 2022 of singleton pregnancies at the Medical School of Hannover, Germany was performed. Retrospective data of 16,092 women who gave birth were used. We examined breastfeeding in our maternity unit (Ever breastfed, Infant formula only, Breastfeeding cessation before discharge, Exclusive breastfeeding at the breast, Breastfeeding and expressed breast milk, Breastfeeding and infant formula) in mothers by self-report, which was a routine survey conducted by a breastfeeding and lactation consultant within the framework of the perinatal quality assurance initiative. During the data collection process, our center collected data analogous to a “baby-friendly hospital” certified by the World Health Organization (WHO) [19]. This certification necessitates a comprehensive analysis of the breastfeeding behaviors of the mothers who have given birth. The definitions pertain to the various forms of breastfeeding as follows:

Ever breastfed: The group of women who ever breastfed includes all women who initiate breastfeeding from the moment they give birth.

Infant formula only: Refers to newborns who were exclusively fed artificial food from birth on.

Breastfeeding cessation before discharge: Refers to women who initially attempted breastfeeding but subsequently transitioned to artificial feeding.

Exclusive breastfeeding at the breast: Women in this group are those who did not transition to artificial feeding at any point.

Breastfeeding and expressed breast milk: The group of women who breastfeed and simultaneously express milk are those who encounter difficulties with direct breastfeeding and supplement with expressed milk. This group is further subdivided into women who breastfeed without medical indication (e.g. because the child is not yet satisfied) and women who breastfeed with medical indication (e.g. because the child has a low weight).

Breastfeeding and infant formula: Women who breastfeed and simultaneously provide artificial milk (e.g. because there is not enough breast milk available).

The data collected relates exclusively to the period of inpatient care directly after the birth until discharge home. Nurses and lactation consultants complete a breastfeeding documentation form for each patient, which was then transferred to an online documentation system.

The Survey also collects data on: Maternal age at beginning of pregnancy, BMI, parity, gestational age at delivery, infant weight at birth and mode of delivery. Inclusion criteria were all singleton pregnancies with live-born singletons. The patient characteristics of cohort I is shown in Table 1. (II) Second a retrospective data analysis of 4,603 mother-child-pairs from 2021 to 2023 of live born, singleton full-term newborns and their mothers at our maternity unit was performed. As part of the inpatient stay data on breastfeeding behavior (Exclusive breastfeeding at the breast, Breastfeeding and expressed breast milk or infant formula, Infant formula only) was collected by self-report. In addition to the characteristics of Cohort I, the following parameters were recorded in this smaller cohort, to examine the prevalence of parameters in relation to breastfeeding behaviour: Maternal BMI, Gestational Diabetes, Diabetes mellitus, Integration problems, Social impacts, Metal stress, Abuse, Underlying health conditions, Long-term medication, Infertility treatment, Rapid pregnancy rate. The patients characteristics of cohort II is shown in Table 2.

**Table 1:** Descriptive statistics of women who gave birth between 2017 and 2022; *N* = 16,092 (Cohort 1)

Characteristics	2017	2018	2019	2020	2021	2022
**Live births** (n)	2835	2986	2999	2824	2856	2624
**Age in years** (mean; st.dev.)	32.0 ± 5.3	32.1 ± 5.3	32.2 ± 5.4	32.3 ± 5.3	32.4 ± 5.3	32.2 ± 5.7
**BMI in kg/m**^**2**^ (mean; st.dev.)	25.1 ± 6.2	25.0 ± 5.8	25.1 ± 5.7	25.7 ± 6.9	25.5 ± 5.9	25.8 ± 7.3
**Gravidity** (mean; st.dev.)	2.2 ± 1.5	2.2 ± 1.4	2.3 ± 1.5	2.3 ± 1.5	2.3 ± 1.5	2.3 ± 1.7
**Parity** (mean; st.dev.)	1.7 ± 1.1	1.7 ± 1.0	1.3 ± 1.2	0.9 ± 1.1	0.9 ± 1.1	0.9 ± 1.2
**Duration of pregnancy in weeks**						
**< 34 + 0** (in %)	6.5	6.5	7.3	3.8	4.1	4.3
**34 + 0–36 + 6** (in %)	15.4	15.3	16.2	8.7	8.3	8.4
**37 + 0–40 + 6** (in %)	77.8	78.1	76.4	78.4	77.7	78.8
**> 41 + 0** (in %)	0.2	0.1	0.1	9.1	9.9	8.5
**Infant weight**						
**Infant weight in gramm** (mean; st.dev.)	3258 ± 676	3254 ± 694	3257 ± 688	3241 ± 707	3252 ± 673	3230 ± 673
**< 2000 g** (in %)	5.3	5.4	5.0	6.2	5.1	5.6
**2000–2499 g** (in %)	5.8	5.4	5.9	5.8	6.2	6.4
**2500–4000 g** (in %)	82.4	82.8	82.0	77.9	79.6	79.1
**> 4000 g** (in %)	6.5	6.4	7.1	10.1	9.1	8.8
**Birth mode**						
**Spontaneous delivery** (in %)	64.9	64.3	65.6	65.9	64.7	61.4
**Vaginal operative delivery** (in %)	3.4	5.4	5.2	6.0	5.5	6.5
**Cesarean section** (in %)	31.7	30.3	29.2	28.1	29.8	32.1

**Table 2:** Descriptive statistics of women who gave birth between 2021 and 2023; *N* = 4,603 (Cohort 2)

Characteristics	
**Age in years** (mean; st.dev.)	32.1 ± 5.6
**BMI in kg/m**^**2**^ (mean; st.dev.)	25.6 ± 6.6
**Gravidity** (mean; st.dev.)	2.3 ± 1.7
**Parity** (mean; st.dev.)	0.9 ± 1.1
**Duration of pregnancy in weeks** (mean; st.dev.)	39.6 ± 1.1
**Infant weight in gramm** (mean; st.dev.)	3424.7 ± 460
**Spontaneous delivery** (in %)	67.4
**Vaginal operative delivery** (in %)	7.1
**Cesarean section** (in %)	25.5
**Maternal days until discharge** (mean; st. dev.)	2.7 ± 2.0
**Child days until discharge** (mean; st. dev.)	2.6 ± 1.4

Before collecting the data, the ethics committee was asked to evaluate the study (No. 11203_BO_K_2024). All methods were carried out in accordance with relevant guidelines and regulations.

### Standard operation procedure for promoting breastfeeding

In our institution, the welfare of women postpartum is overseen by nurses and pediatric nurses, who are obliged to undergo annual training in breastfeeding counselling. In addition, the institution employs certified breastfeeding and lactation consultants (IBCLC) exclusively for the purpose of breastfeeding counselling. The standard operating procedure (SOP) of the clinic clearly regulates breastfeeding instructions and care. The approach adopted in breastfeeding instruction and support for mothers aligns with the recommendations of the World Health Organization’s Baby-friendly hospital initiative [19]. Interventions that serve as support at our clinic include breastfeeding instructions during pregnancy, during the inpatient stay, and further care from midwives in private practice after discharge. In the event of breastfeeding complications, group sessions or consultations with a breastfeeding consultant via a designated breastfeeding hotline are available even after discharge.

### Definitions and variables

The definition of special outcome parameters are as follows: According to the WHO definition, BMI was calculated from maternal body weight and height measured at the first antenatal visit. Five BMI groups were defined (normal weight: BMI from 18.5 to < 25 kg/m^2^, overweight: BMI from 25 to < 30 kg/m^2^ obesity grade I: BMI from 30 to < 35 kg/m^2^, obesity grade II: from 35 to < 40 kg/m^2^ and obesity grade III: BMI ≥ 40 kg/m^2^).

The current classification for Gestational Diabetes and Diabetes mellitus are taken from the current guideline for gestational diabetes and the classification for diabetes mellitus from the German Diabetes Society.

Integration problems or economic burdens count as social impacts. Cultural discrepancy was defined as a cultural or strong language barrier so that breastfeeding instruction could not be adequately implemented. Mental stress was defined as depressive disorders or anxiety disorders during pregnancy. Abuse is the dependence on a substance such as nicotine or drugs.

Underlying health conditions represents all types of conditions requiring treatment. The most common interventions were the treatment of endocrinological diseases, cardiovascular diseases, internal diseases, glucose metabolism disorders like hydramnios, fetal hypertrophy and Diabetes mellitus or Gestational diabetes as well as preeclampsia and intrauterine growth restriction. Long-term medication was defined as medication started before the beginning of pregnancy and continued after delivery.

Infertility treatment describes any type of invasive and non-invasive support to get pregnant. Rapid pregnancy rate describes a new occurred pregnancy after a previous delivery within one year.

### Statistical analysis

Data was assembled in a databank and analyzed using Microsoft Excel 2021 (version 16.56; Microsoft Corp., Redmond, WA, USA). Statistical analyses were performed using Graph pad prism 9 software (GraphPad Software Inc.) and IBM SPSS Statistics 28 (IBM SPSS Software). Shapiro-Wilk normality test was used to test for normal distribution of epidemiological data. Unpaired t test and Mann Whitney test were used as appropriate. Comparisons between groups were performed using one-factor analyses of variance for continuous variables and χ2 tests for categorical variables. Missing values were not replaced. The significance level was set at *p* < 0.05.

## Results

### Development of breastfeeding from 2017 to 2022 (Cohort 1)

Between 2017 and 2022, 16,092 patients gave live birth to a singleton at the Medical School of Hannover. The mean age of the women during the years of observation was between 32.0 and 32.4 years. The BMI at the start of pregnancy was on average between 25.0 kg/m^2^ and 25.8 kg/m^2^. The mean gravidity of the subjects was 2.3, the mean parity was 0.9. Table 1 shows the distribution of births over the years regarding gestational age, birth weight and mode of delivery.

We examined breastfeeding in our maternity unit from 2017 to 2022 in mothers by self-report (groups: Ever breastfed, Infant formula only, Breastfeeding cessation before discharge, Exclusive breastfeeding at the breast, Breastfeeding and expressed breast milk, Breastfeeding and infant formula).

In 2017, the proportion of women who had ever breastfed was 93%. This rate decreased steadily to 83% by 2022. While in 2017 78% of all women who gave birth exclusively breastfed at the breast, in 2022 this was only 57% of all women.

The rate of women who feed infant formula only remained stable at between 5 and 10% over the observed period. The rate of women who ceased breastfeeding before being discharged remained at a very low level, at approximately 1%.

The rate of women who fed their children with breast milk in addition to breastfeeding, whether with or without a medical indication, increased slightly over the observation period from 0% in 2017 to 8% in 2022.

Breastfeeding and simultaneous feeding with infant formula increased over time from 8% of all births in 2017 to 28% of all births in 2022.

**Fig. 1:**
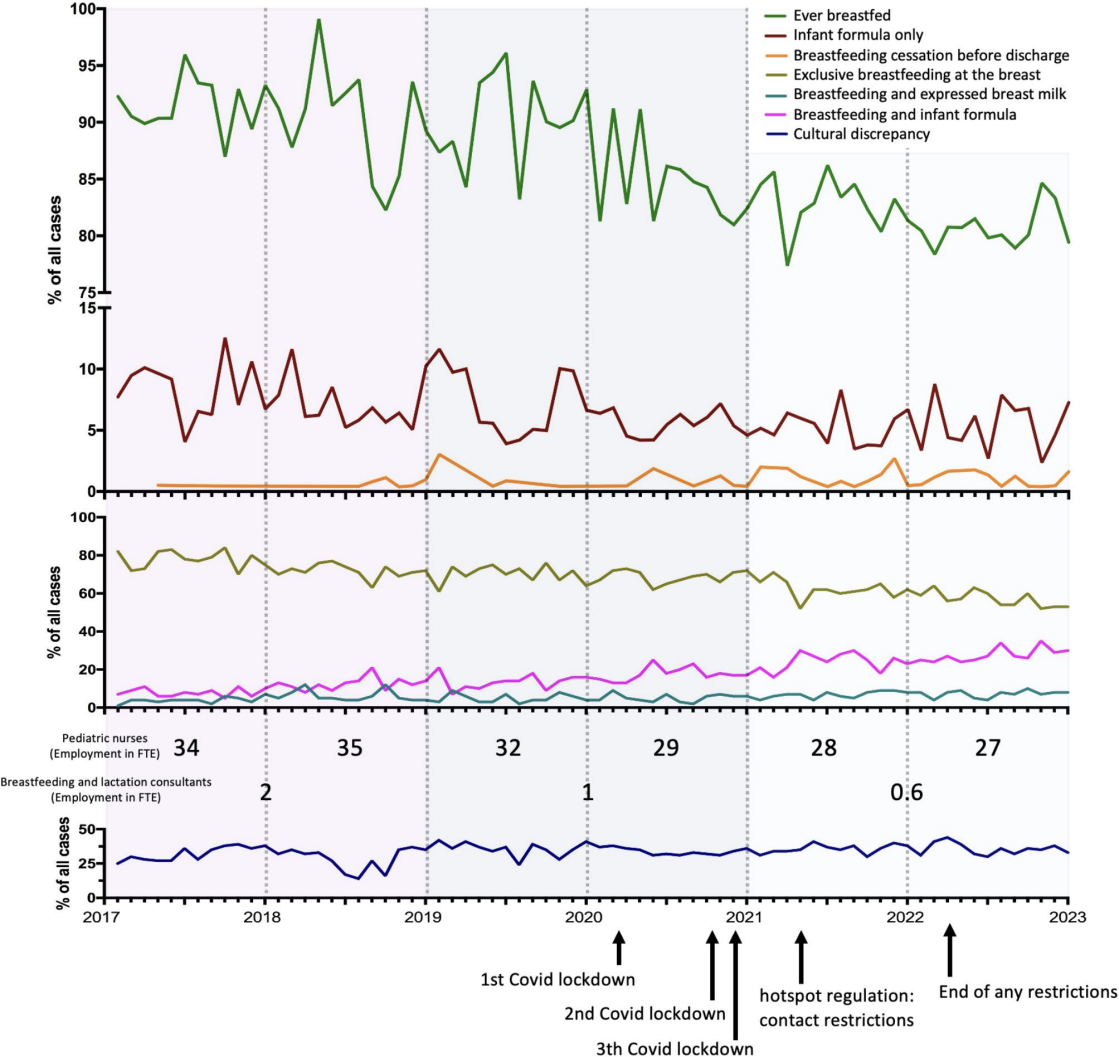
Incidence of types of feeding of the newborn and development of the clinic structure from 2017 to 2023

The number of births from 2017 to 2022 in our center is declining slightly (number of births year: n: 2017: 2,918; 2018: 2,907; 2019: 2,986; 2020: 2,824; 2021: 2,856; 2022: 2,624). The number of pediatric nurses (expressed as a percentage of employment) decreased. While in 2017 1.17 pediatric nurses were employed per 100 births, in 2022 1.03 pediatric nurses were employed per 100 births. In 2017 there were 0.07 breastfeeding and lactation consultants per 100 births, and in 2022 there were 0.02 breastfeeding and lactation consultants per 100 births (Fig. 1).

### Development of employed pediatric nurses and breastfeeding and lactation consultants

The number of pediatric nurses and breastfeeding and lactation consultants was recorded as one full-time employee is 1 full-time equivalent (FTE). Therefore, 1 corresponds to one full-time employee in full-time employment. At the initiation of data collection in 2017, 34 FTE of the pediatric nursing workforce and 2 FTE of breastfeeding and lactation consultancy personnel were in employment. However, by 2023, the number of pediatric nurses had decreased to 27 FTE. The number of breastfeeding and lactation consultants fell to 1 FTE in 2019 and to 0.6 FTE of a full-time employee in 2021. In relation to the number of live births per year 1.17 pediatric nurses and 0.07 breastfeeding and lactation consultants were employed per 100 births in 2017. In 2022 1.03 pediatric nurses and 0.02 breastfeeding and lactation were employed per 100 births.

### The relationship between feeding types and specific patient characteristics (Cohort 2)

Table 2 shows the retrospective data analysis of 4,603 mother-child-pairs from 2021 to 2023 of live born, singleton full-term newborns and their mothers at our maternity unit. Mean maternal age was 32.1 years, body mass index at the beginning of pregnancy was 25.6 kg/m^2^ and fetal birth weight was 3424.7 g. Gestational age at delivery was 39.6 weeks of gestation.

### BMI, gestational age and parity

To examine the influence of specific maternal characteristics a retrospective data analysis of the mother-child-pairs was done.

First-time mothers breastfed exclusively in 74.1% of cases. Women giving birth to their second child breastfed exclusively in 76.3% of cases. Third- and fourth-time mothers breastfed slightly less frequently, at 70.4% and 71.7% respectively, while women giving birth to their fifth or any subsequent child (higher multiparae) breastfed exclusively in 61% of cases. Across all parities, the proportion of women who supplemented their feeding in addition to exclusively breastfeeding was approximately the same (17.1–22.2%). The exceptions were higher multiparae, who supplemented their feeding in addition to exclusively breastfeeding in 27.4% cases.

The rate of exclusive breastfeeding was highest in the group of women who gave birth between 41 + 0 and 42 + 0 weeks of gestation (78.2%). The rate of infant feeding with formula only was highest in the group of infants delivered between 37 + 0 and 38 + 0 weeks of gestation (10.1%).

**Fig. 2:**
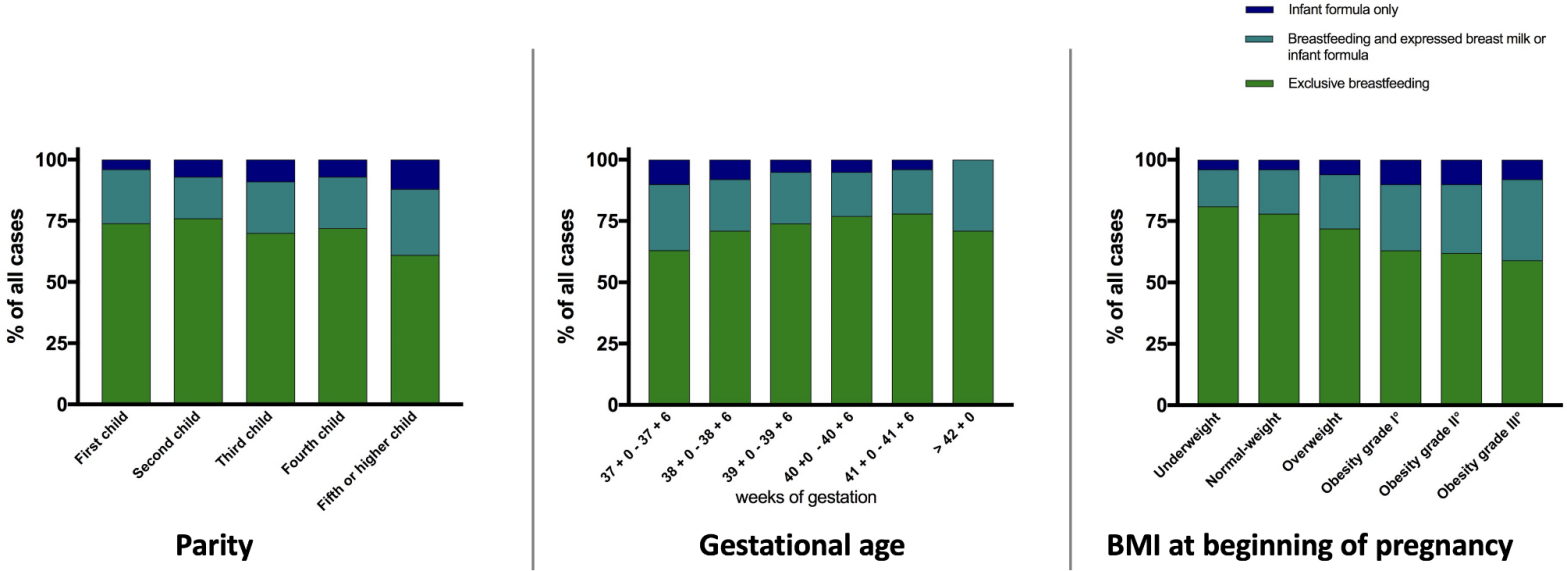
The relationship between types of feeding of the newborn and BMI, gestational age and parity

Women with a normal BMI breastfed 78.2% of their newborns exclusively, 4.3% fed formula only and 17.5% fed alongside normal breastfeeding. The higher the BMI, the lower the rate of exclusively breastfeeding mothers. Only 59.1% of women with grade 3 obesity exclusively breastfed their child. 32.7% were breastfeeding and supplementary feeding and 8.2% were exclusively feeding formula (Fig. 2). Similar results were obtained when considering BMI at birth in relationship to types of breastfeeding (Supplemental Fig. 1).

### Breastfeeding and birth mode

78.1% of all women who gave birth spontaneously breastfed exclusively. 17.1% supplemented their feedings in addition to breastfeeding, 4.8% fed formula only. 69.5% of women who gave birth by assisted vaginal birth breastfed exclusively and 26.2% supplemented their feedings. Exclusive formula feeding occurred in 4.3% of cases.

**Fig. 3:**
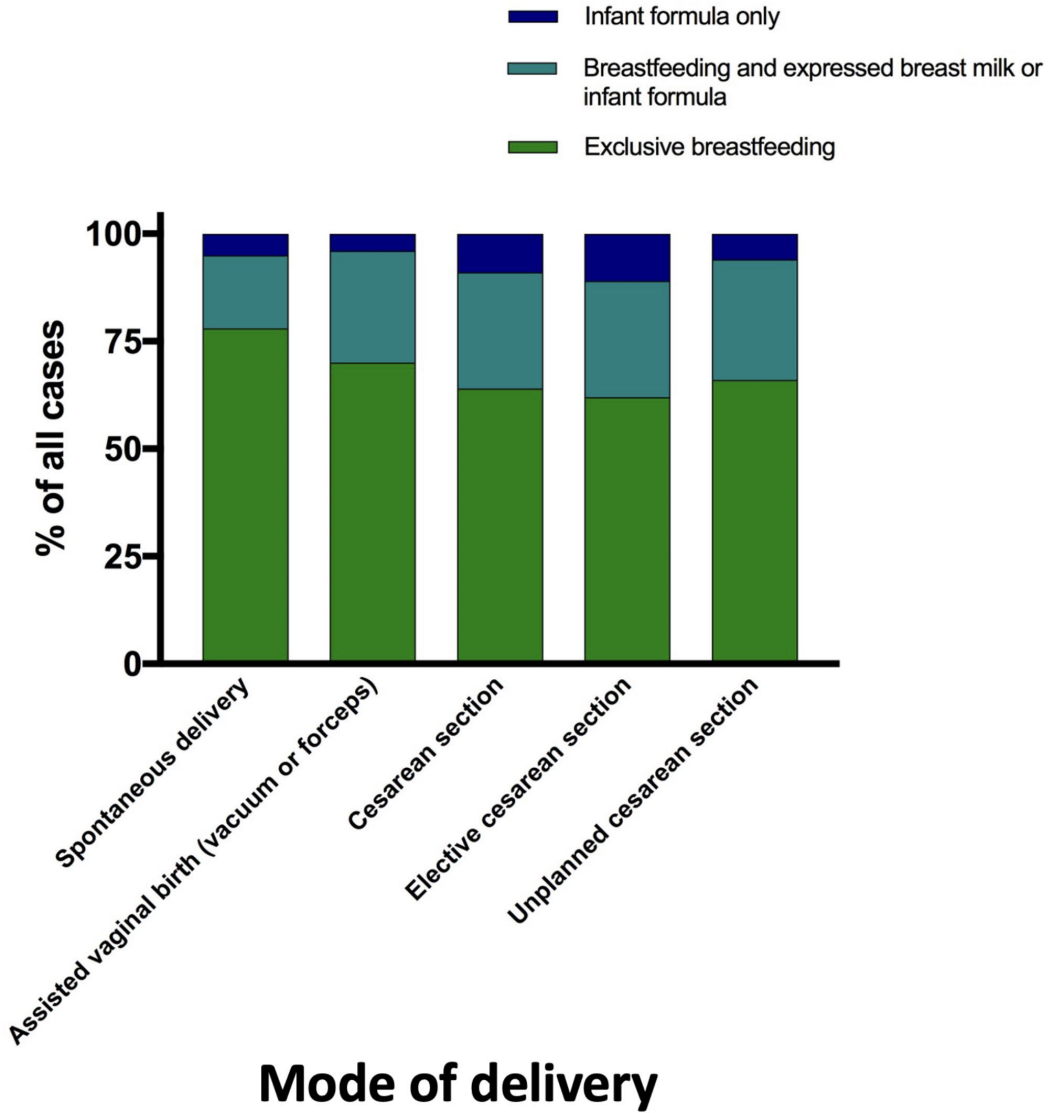
The relationship between types of feeding of the newborn and mode of delivery

Women who gave birth by caesarean section breastfed in 63.7% (elective caesarean section: 62.3%; unplanned caesarean section: 66.1%). Supplementary feeding was used in 27.2% (elective cesarean section: 26.9%; unplanned cesarean section: 27.6%), formula only feeding was used in 9.1% of cases and was highest in the elective cesarean section group (elective cesarean section: 10.8%; unplanned cesarean section: 6.3%) (Fig. 3).

### Breastfeeding and special risk factors

There were no differences in breastfeeding behavior of the mothers regarding maternal age over 35, infertility treatment or rapid pregnancy rate.

Mothers with underlying health conditions (not present vs. present: Exclusive breastfeeding (EBF): 75.2% vs. 68.6%, Breastfeeding and substitution (BFS): 19.7% vs. 22.5%, formula substitutes (S): 5.1% vs. 9%; *P* = 0.001), long-term medication (EBF: 74.4% vs. 64.0%, BFS: 19.9% vs. 26.7%, S: 5.7% vs. 9.3%; *P* = 0.001) and diabetes, both diabetes mellitus (EBF: 74.2% vs. 62.0%, BFS: 20.0% vs. 29.5%, S: 5.8% vs. 8.5%; *P* = 0.008) and gestational diabetes (EBF: 74.5% vs. 65.9%, BFS: 19.8% vs. 26.6%, S: 5.7% vs. 7.5%; *P* = 0.002), breastfed significantly less frequently.

**Fig. 4:**
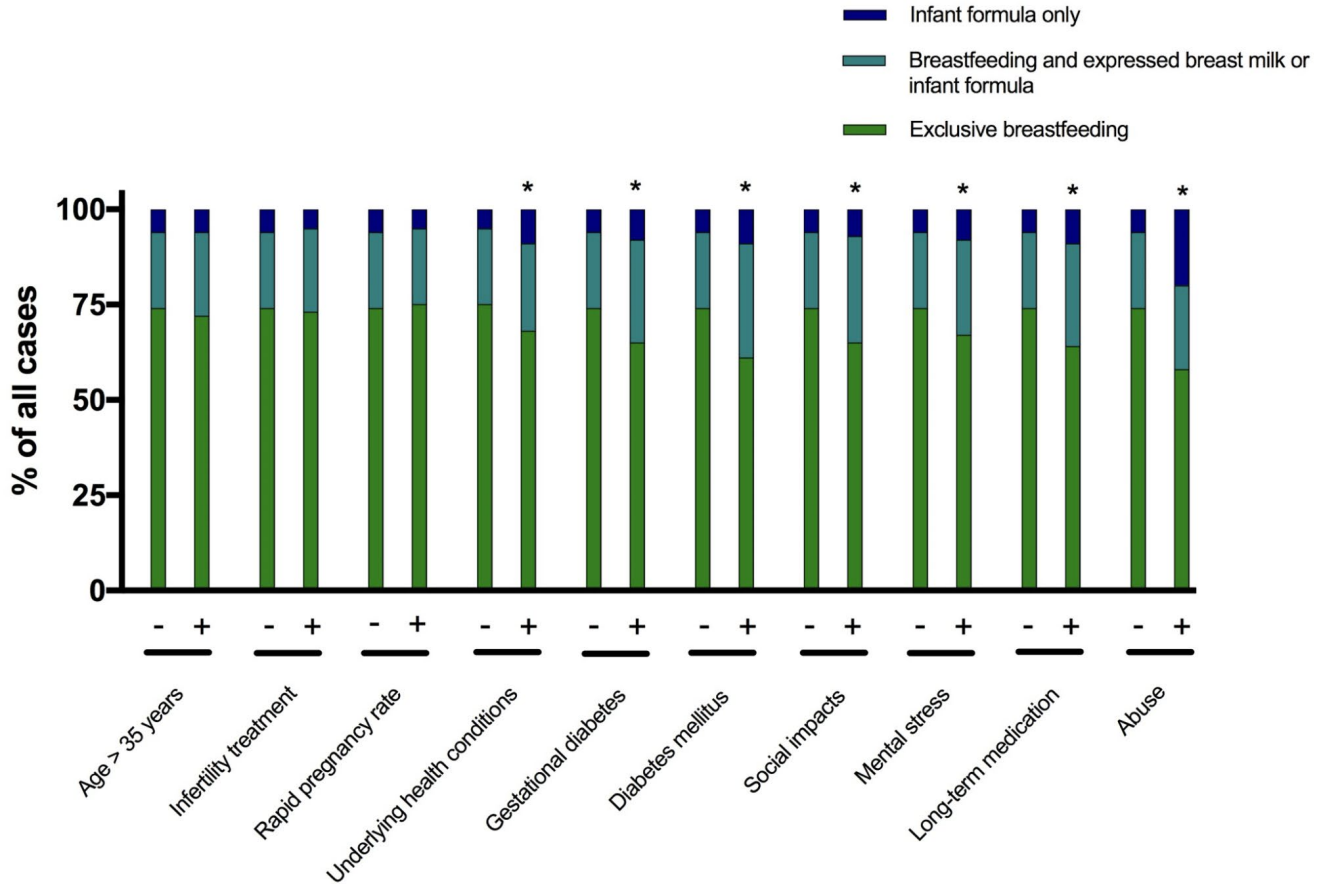
The relationship between types of feeding of the newborn and special risk factors

Social impacts (EBF: 74.5% vs. 65.3%, BFS: 19.7% vs. 27.8%, S: 5.8% vs. 6.9%; *P* = 0.045) and mental stress (EBF: 75.2% vs. 68.6%, BFS: 19.7% vs. 22.5%, S: 5.1% vs. 9.0%; *P* = 0.001) also led to a lower breastfeeding rate (Fig. 4).

## Discussion

Our study showed that the rate of ever breastfeeding women started at 93% in 2017 and decreased by 10–83% in 2022. The rate of exclusively breastfeeding women decreased by 21%. While the group of feeding infant formula only and breastfeeding cessation before discharge remained stable, the rate of supplementary feeding, and breastfeeding and feeding infant formula, increased significantly.

In recent years, several studies have been conducted in Germany to investigate breastfeeding behavior. Therefore, a general interest in studies on breastfeeding behavior exists. The results of our study are comparable to the multicentric observational SINA (Breastfeeding in North Rhine-Westphalia) study examined hospital breastfeeding management and maternal breastfeeding behaviors during the COVID-19 pandemic in Germany in 2021/2022 [20]. This survey showed a breastfeeding initiation of 92.2%. Kersting et al. estimated the proportion of any breastfeeding to be 85% on average and the proportion of exclusive breastfeeding to be 67% on average in 2021 in Germany [21]. The results of our study cannot be fully compared with data from other European countries. Breastfeeding is not consistently recorded in most countries. In addition, the research methods and definitions of breastfeeding differ [22]. In the Scandinavian countries with proportion of exclusively breastfed infants of 90% at 1 month of life, initial breastfeeding rates appear to be higher than those we found in our unit [23]. In Italy, initial breastfeeding rates are comparable to those we collected (91.1%), whereas in the Netherlands, initial breastfeeding rates appear to be lower than those we collected (78.0%) [24, 25].

COVID-19 disease is thought to have an impact on breastfeeding rate. The decrease in breastfeeding rates was observed to occur more strongly in parallel with the onset of the COVID-19 pandemic in our center. At the onset of the pandemic (March 2020), a general policy of no visiting was implemented in our clinic, with fathers permitted to visit for a limited period of five hours a day. Subsequently, from September 2022, an additional individual was permitted to visit. From March 2023, a relaxation of the visitor policy was implemented, with fathers permitted to visit for a duration of eight hours. However, from August 2023, the pre-pandemic visitor regulations were reinstated.

It has been hypothesized that the modified visitor policies may have facilitated increased opportunities for women to breastfeed in a more tranquil environment, thereby contributing to an augmentation in breastfeeding rates. However, this assumption has not been substantiated. The limitation of close contact with healthcare professionals, a factor that has been shown to promote breastfeeding behavior, during the pandemic period is noteworthy. The limitations imposed by contact restrictions and isolation measures resulted in a significant reduction in the provision of breastfeeding advice, or even its complete cessation. This phenomenon has been documented in other centers as well [26]. We assume that the effects of increased time to attempt breastfeeding without distraction from visitors could not offset the effect of poorer breastfeeding support.

We observed that the number of pediatric nurses available to care for the mothers, as well as the number of breastfeeding and lactation consultants, decreased in relation to the care per 100 births over the observed period. Insufficient milk production was cited as the main problem by mothers who intended to breastfeed but had not started. Insufficient breast milk is a common reason for early weaning [6, 27]. Additional assistance is required to assist mothers who encounter difficulties with breastfeeding and who intend to breastfeed in order to overcome these difficulties and to enable them to continue breastfeeding. A Cochrane review evaluated studies on breastfeeding promotion interventions and found that any form of breastfeeding promotion leads to a longer duration of all types of breastfeeding [28]. Difficulties in starting breastfeeding can lead to early weaning. Appropriate advice from trained staff can help to avoid this. If exclusive breastfeeding is not possible, the mother should be encouraged to breastfeed the child at least partially, as this also benefits the child’s health. The decreasing rate of clinically trained professionals may be one reason for the decline in primarily breastfeeding women in our center. The reasons for the decline in consultants are varied. The decrease in the number of breastfeeding counsellors available on the labor market may be due to the profession becoming less attractive, resulting in a decline in women wishing to enter or remain in the profession. This may be due to a lack of recognition, difficult working conditions, limited training opportunities and changing social structures [29, 30]. It is imperative that a robust lobbying effort is maintained to ensure the profession receives adequate recognition and support. The decline in the number of lactation consultants at our facility has resulted in an increased workload for the remaining consultants and a less attractive working environment. Our analysis reveals that the decrease in lactation consultants has coincided with an increase in the prevalence of women who are using artificial feeding methods in addition to breast milk. Consequently, there is a necessity to enhance the appeal of the working environment for prospective applicants, with a view to facilitating superior care for mothers.

Women giving birth to their second child breastfed exclusively in most cases. As parity continued to increase, willingness to breastfeed decreased. It is important to consider lactation history. Bad breastfeeding experiences with the first child have often led to the decision to decide against breastfeeding from the outset [6].

Women with a normal BMI breastfed 78.2% of their newborns exclusively. The higher the BMI, the lower the rate of exclusively breastfeeding mothers. Only 59.1% of women with grade 3 obesity exclusively breastfed their child. The lower incidence of breastfeeding in women with a high BMI is multifactorial. The occurrence of pre-existing conditions such as diabetes or hypertension, the presence of physical difficulties associated with breastfeeding, and the influence of psychological factors such as disrupted body image have all been identified as potential factors that contribute to this issue [31]. The impact of BMI, parity and maternal age on breastfeeding behavior has been previously documented in the literature [32, 33]. In the present study, we extend these findings by examining the impact of these factors on breastfeeding behavior within a tertiary obstetric center, which is characterized by a higher proportion of women with potentially selected risk factors.

Mothers who had a spontaneously birth breastfed most frequently. The lowest rate of breastfeeding was observed in the group of women who underwent cesarean section. The lower breastfeeding rate after cesarean deliveries can be explained by the surgical stress, delayed skin-to-skin contact, altered hormonal changes and other types of postpartum support in comparison to vaginal delivery [34, 35].

Mothers with special risk factors like underlying health conditions, diabetes mellitus, gestational diabetes or social impacts and mental stress breastfed less frequently than women who did not have these risk factors. A low socioeconomic status is considered a risk factor for the initiation and duration of breastfeeding in Europe and the USA [36, 37].

### Strengths and limitations

The study was a retrospective data analysis of all births of a non-selected cohort. As data was recorded by self-report, which was a routine survey conducted by a breastfeeding and lactation consultant within the framework of the perinatal quality assurance initiative the data collection bias is considered to be low. The study design comprised a complete cohort of all mothers who gave birth during the observation period, thereby eliminating any potential for selection bias. A limitation of our study is the regional specificity of the data collected. This is due to the unequal framework conditions caused by regional, social and socio-cultural peculiarities. A survey in Bavaria revealed a breastfeeding rate of around 90%, while a different survey showed, that only 74% of infants were initially breastfed in Lower Bavaria at the same time [38].

## Conclusion

Targeted, holistic counselling of the mother during her hospital stay is crucial to improve access to breastfeeding and to prevent breastfeeding problems. Quality assessment and quality assurance can lead to improved outcomes in breastfeeding rates. Changes in hospital practice and adequate guidance and support for the mother can be effective interventions [39]. The role of tertiary obstetric centers in breastfeeding should be to provide informative education, early guidance and support during the inpatient stay, and follow-up support after discharge. Tertiary obstetric centers should serve as a backup institution even after discharge to support the duration of breastfeeding in the long term.

## Electronic supplementary material

Below is the link to the electronic supplementary material.

The relationship between types of feeding of the newborn and maternal BMI at birth

## Data Availability

Data is provided within the manuscript. Raw data were generated at the Department of Obstetrics and Gynecology of Hannover Medical School (MHH). The datasets used and analyzed during the current study are available from the corresponding author upon reasonable request.
