# Correction: Development and prevalence of breastfeeding initiation in a tertiary obstetric center and its influencing factors

**DOI:** 10.1186/s13006-025-00735-3

**Published:** 2025-05-20

**Authors:** A. L. Biermann, L. Steinkasserer, L. Radomsky, C. von Kaisenberg, P. Hillemanns, Lars Brodowski

**Affiliations:** Department of Gynaecology and Obstetrics, Hannover Medical School, Carl-Neuberg-Straße 1, 30625 Hannover, Germany

**International Breastfeeding Journal (2025) 20:24**

10.1186/s13006-025-00717-5

Following publication of the original article, it was brought to the authors’ attention that an incorrect wording in the methods part had been provided. Namely, ‘During the data collection process, our center was certified as a “baby-friendly hospital” by the World Health Organization (WHO).’ has been corrected to ‘During the data collection process, our center collected data analogous to a “baby-friendly hospital” certified by the World Health Organization (WHO).’

The error has since been corrected in the original article. The authors apologize for any inconvenience caused.

