# Supplementary figures and images for: Development and prevalence of breastfeeding initiation in a tertiary obstetric center and its influencing factors
Source: Int Breastfeed J. 2025 Apr 3;20:24. doi: 10.1186/s13006-025-00717-5 (PMC11969701; doi:10.1186/s13006-025-00717-5)

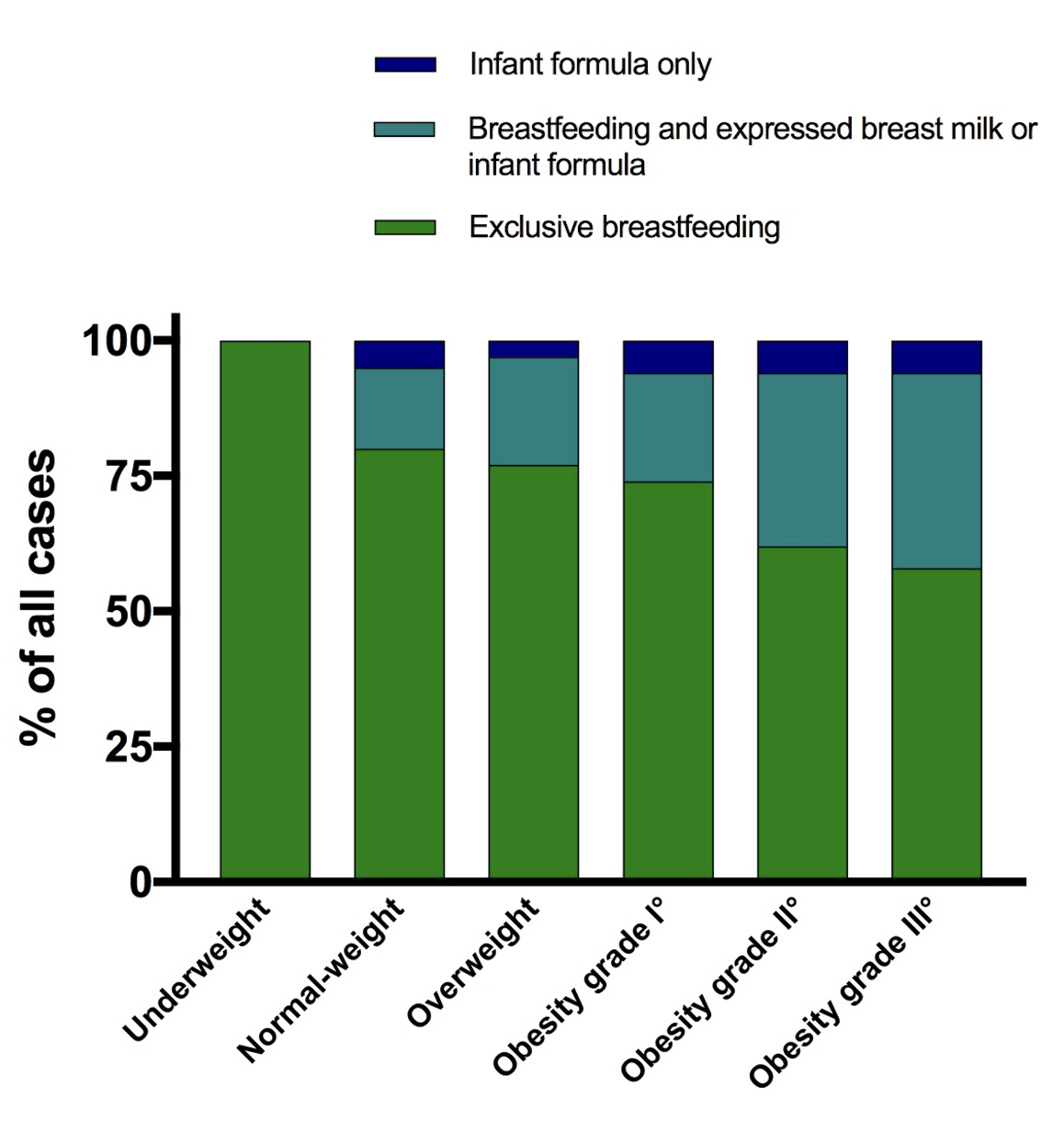

Supplement: Supplementary file 1 — The relationship between types of feeding of the newborn and maternal BMI at birth [file 13006_2025_717_MOESM1_ESM.png]
